# Interleukin-6, -17, and -35 levels in association with clinical status in stage III and stage IV periodontitis: a cross-sectional study

**DOI:** 10.1186/s12903-024-04751-3

**Published:** 2024-08-30

**Authors:** Müge Altaca, Elif Ilke Cebesoy, Necla Asli Kocak-Oztug, Ilknur Bingül, Emine Cifcibasi

**Affiliations:** 1 Faculty of Dentistry, Department of Periodontology, Istanbul University, Istanbul, 34116 Turkey; 2 Institute of Graduate Studies in Health Sciences, Department of Periodontology, Istanbul University, Istanbul, 34126 Turkey; 3 School of Dentistry, Faculty of Health and Behavioural Sciences, The University of Queensland, Brisbane, QLD 4006 Australia; 4 Faculty of Medicine, Department of Medical Biochemistry, Istanbul University, Istanbul, Turkey

**Keywords:** Gingival crevicular fluid, Periodontitis, Inflammation, Interleukin-6, Interleukin-17, Interleukin-35

## Abstract

**Background:**

This study compared the concentrations of interleukin (IL)-6, IL-17, and IL-35 in the gingival crevicular fluid of periodontally healthy participants with individuals who had stage III and IV periodontitis.

**Methods:**

In total, 60 participants with stage III grade B-C (*n* = 12)—stage IV grade C (*n* = 18) periodontitis and 30 healthy controls were included in this cross-sectional study. Full-mouth clinical periodontal measurements were performed. Concentrations of IL-6, IL-17, and IL-35 were determined using enzyme-linked immunosorbent assays. Parametric/nonparametric methods, Pearson’s/Spearman’s correlation, and logistic regression methods were used for data analyses.

**Results:**

The periodontitis group exhibited significantly higher levels of IL-6, IL-17, and IL-35 compared with the healthy group (*p* < 0.001). IL-17 levels had a positive correlation with pocket depth (PD) (r = 0.395; *p* = 0.031) in the periodontitis group. IL-6, IL-17, and IL-35 levels were associated with periodontitis (odds ratio [OR] = 1.344, 95% confidence interval [CI] = 1.159–1.56; OR = 1.063, 95% CI = 1.025–1.102; OR = 1.261, 95% CI = 1.110–1.434, respectively) (*p* < 0.001, *p* = 0.001, *p* < 0.001, respectively). Full-mouth and sampling sites PD and clinical attachment loss (CAL) values were significantly higher in the periodontitis group than in the healthy group (*p* < 0.001).

**Conclusions:**

This study revealed upregulated levels of IL-6, IL-17, and IL-35 in periodontitis patients compared to healthy individuals. IL-17 shows a correlation with increased PD. These findings suggest a potential association between these cytokines and severe and advanced periodontitis.

**Trial registration:**

The trial was registered in ClinicalTrials.gov with this identifier NCT05306860 on 24/01/2022.

**Supplementary Information:**

The online version contains supplementary material available at 10.1186/s12903-024-04751-3.

## Introduction

Periodontitis, characterized by the progressive degeneration of the tooth-supporting structure, is a chronic multifactorial inflammatory disease linked to dysbiotic plaque biofilms. The immune response plays a pivotal role in determining the course of disease. Periodontitis is characterized by gingival bleeding, periodontal pockets, clinical attachment loss (CAL), loss of alveolar bone, and diminished periodontal tissue support [1].

The 2017 global workshop on the Classification of Periodontal and Peri-Implant Diseases and Conditions introduced a new periodontitis grading and staging system. Because periodontal diseases are multifactorial, the new classification system provides a more comprehensive approach for disease classification and staging [2]. An additional component of this workshop included the incorporation of a precise definition of periodontal health. According to definition, periodontal health is characterized by the absence of inflammation detectable through clinical examination, and at a biological level, there's a mechanism of immune surveillance that supports the maintenance of healthy gingiva and overall oral cavity balance [3].

Periodontal disease initiation requires exposure to pathogens; however, the host immune response and cytokine profile appear to dominate subsequent disease progression [4, 5]. Cytokines coordinate various types of immune cells and mediate a broad range of physiological processes, including target cell development, growth, and differentiation [6, 7]. Pathogen-produced endotoxins trigger the production of cytokines by host immunological inflammatory cells, comprising macrophages and Th cells [7, 8]. Concerning the clinical significance of biomarkers in the current classification, it is anticipated that biomarkers of the immune system, in conjunction with radiographic and clinical data, can be utilized as supplementary information in clinical evaluations [9].

Several studies have demonstrated the proinflammatory role of interleukin (IL)-6 in the context of periodontitis [10–12]. The production of this cytokine has been documented in a range of cell types, such as endothelial cells, fibroblasts, and monocytes [13]. Proinflammatory biomarkers, including tumor necrosis factor (TNF)-α, IL-1, and IL-6, can stimulate osteoclasts, which are critical molecules in the regulation of periodontal inflammation-associated immune responses [13, 14].

IL-17 is a proinflammatory cytokine generated by CD4 + T cells (T helper 17 [Th17] cells). IL-17A to F are members of the IL-17 family [15]. Elevated concentrations of IL-17A have been identified in the serum, gingival crevicular fluid (GCF), and saliva of individuals with periodontitis [15–19]. It has been proposed that IL-17 induces bone resorption and inflammation by inducing chemokine release; stimulating the production of IL-6, TNF-α, and IL-1β; and inducing various matrix metalloproteinases [15, 20]. Additionally, clinical trials have demonstrated that individuals with periodontitis exhibit significantly elevated concentrations of IL-6, a critical signaling molecule that facilitates Th17 differentiation, compared with the control group [21, 22]. The potent osteoclastogenic properties of IL-17 are predominantly attributed to its capacity to induce osteoblasts to generate Receptor Activator of Nuclear factor-κ B Ligand (RANKL) expression. By co-expressing RANKL and IL-17, Th17 cells can function as a specialized subset of osteoclasts; thus, T-cell activation has been associated with inflammatory bone degradation [23, 24].

IL-35 is a relatively new component of the IL-12 cytokine family, which includes three other variants: IL-27, IL-23, and IL-12 [25]. IL-35 functions as an inhibitory factor in chronic inflammation, autoimmune diseases, and various immune disorders. Moreover, immune inhibition capacity is correlated with the secretion of IL-35 from Treg cells [26]. IL-35 impedes Th17 cell activation, inhibiting IL-17 production [27]. In summary, it has been suggested that IL-35 facilitates the modulation of Th17-related chronic inflammatory diseases, including periodontitis [28]. The exact mechanisms by which IL-35 influences Th17 cell activation and subsequent IL-17 production remain unknown. In recent studies, compared with healthy individuals, the concentrations of IL-17 and IL-35 in GCF were substantially increased with chronic periodontitis (CP) [7, 27, 29]. In contrast, another study showed that the healthy group exhibited a considerably higher concentration of IL-35 in GCF, compared with participants who had CP and gingivitis [30].

The interactions of IL-6, IL-17, and IL-35 are hypothesized to potentially play roles in common pathways in the pathogenesis of periodontal disease. There exists a paucity of studies exploring the correlation between these three cytokines and the current classification of periodontitis. This study's main objective was to compare the concentrations of IL-6, IL-17, and IL-35 in the GCF of individuals with Stage III and IV periodontitis and healthy controls. Our secondary objective was to assess the associations between biomarkers and clinical parameters in the abovementioned groups. Furthermore, biochemical and clinical data from subgroups of stage III and IV periodontitis and healthy individuals were compared.

## Materials and methods

### Study population and design

This cross-sectional study enrolled 60 participants (30 periodontally healthy participants and 30 individuals with periodontitis stage III grade B-C [*n* = 12]—stage IV grade C [*n* = 18]) who presented to Istanbul University (IU), Faculty of Dentistry (FD), Department of Periodontology from April 2022 to June 2023, based on initial clinical and radiographic evaluations (aged 18–65 years). The protocols in the present study were confirmed by the Istanbul University Faculty of Dentistry Ethics Committee (Approval no: 2022/679620, file number:2021/71), and the methods were performed in accordance with the Helsinki Declaration guidelines (version Nov. 2013). This study was registered on ClinicalTrials.gov (NCT05306860). Prior to study initiation, written informed consent was collected from each participant. The control group consisted of healthy individuals who exhibited less than 10% bleeding sites with probing depths ≤ 3 mm and showed no signs of attachment or bone loss, as per the 2017 definition of periodontal health. The periodontitis group comprised participants who matched the following inclusion criteria: ≥ 20 teeth, no systemic disease, no smoking history, no antibiotics within the previous 6 months, no regular medications, no recent periodontal therapy, no ongoing pregnancy or breastfeeding, and a diagnosis of stage III–IV grade B–C periodontal disease according to the 2017 classification. The periodontitis group included individuals diagnosed with S III and S IV periodontitis, characterized by detectable interdental clinical attachment loss (CAL) of ≥ 5 mm at non-adjacent teeth, along with radiographic evidence of bone loss extending to the middle or apical third of the root, according to the 2017 definition of periodontitis.

### Clinical periodontal measurements

Six distinct measurements were taken for each tooth (mesiolingual, mid-lingual, distolingual, mesiobuccal, mid-buccal, and distobuccal), excluding wisdom teeth, using a Williams-type periodontal probe (Hu-Friedy, USA) with guided (0.25 N) force by the same calibrated researcher (MA). Recorded periodontal parameters were as follows: gingival index (GI) [31], plaque index (PI) [32], clinical attachment loss (CAL), pocket depth (PD), bleeding on probing (BOP) [33], and tooth mobility (MOB) [34]. To assess tooth mobility, the tooth was lightly tapped in a buccal-lingual direction using dental instruments, and its movement relative to adjacent teeth was observed. Additionally, intrudability was evaluated by applying pressure in an apical direction [34, 35].

### GCF sampling

In the periodontitis group, GCF was sampled from the six deepest active periodontal pockets. In the control group, GCF was sampled from six non-inflamed sites, including incisors, premolars, and molars, selected from non-adjacent teeth on their buccal and lingual surfaces. GCF samples from both groups were collected 1 week after clinical measurements. Cotton pads were used to isolate GCF sample collection sites. Subsequently, dental plaque was removed from the corresponding tooth with a cotton pellet, and a gentle dry technique was implemented from a distance of 20 cm. Next, standard absorbent 2 × 8 mm paper strips (Periopaper®, Proflow Inc., Amitiyville, NY, USA) were positioned in the gingival sulcus of periodontally healthy participants or the periodontal pockets of periodontitis participants until mild resistance was detected, then kept in place for 30 s. Six strips were obtained from all participants, pooled in an Eppendorf tube, and stored at − 80 °C (New Brunswick Scientific Ultra-Low-Temperature Freezer) until laboratory analyses. In cases of bleeding during sample collection, the paper strips were discarded.

### IL-6, IL-17, and IL-35 analyses

Enzyme-linked immunosorbent assays (ELISA) were used to measure IL-6, IL-17A, and IL-35 concentrations in GCF samples at the IU Faculty of Medicine, Department of Medical Biochemistry. On the day of analysis, 20 min were allotted for the Eppendorf tubes each with 6 periopapers to reach room temperature. A volume of 450 µl of a buffer solution consisting of 1% bovine serum albumin (BSA)-phosphate-buffered saline (PBS) plus Tween (pH: 7.4) was introduced into the Eppendorf tubes, then incubated on a shaking platform for elution overnight at 4 °C. The standards and samples from both healthy subjects and those with periodontitis were pipetted into microplate wells. Absorbances at 450 nm were read using a spectrophotometer (Biotech, UK). Standard concentrations and absorbances were utilized to calculate IL-6, IL-17, and IL-35 concentrations (pg/mL) in samples from both healthy and periodontitis groups. Subsequently, the samples were analyzed by ELISA according to the manufacturer's instructions, employing commercially available reagents (IL-6, IL-17A, and IL-35 human kits, MyBioSource, San Diego, CA, USA).

### Statistical analyses

Power analysis in this study was conducted using G*Power version 3.1.9.6, based on the IL-35 levels in GCF from the reference article by Köseoğlu et al. [30] a 95% confidence level (1-α), a 95% test power (1-β), and an effect size of d = 1.055. The analysis showed that 25 participants should be enrolled in each group according to the two-way hypothesis. To compensate for possible dropouts, 60 participants were included in this study, comprising 30 healthy controls and 30 participants with periodontitis.

IBM SPSS V23 and AMOS V24 were used for data analysis. The Shapiro–Wilk test was used to assess the normality of the data distribution. For binary groups, Independent Two-sample *t* tests were used to analyze normally distributed data, whereas the Mann–Whitney U test was used for non-normally distributed data. The Kruskal–Wallis and Dunn tests were used to compare non-normally distributed data among three or more groups. One-way analysis of variance (ANOVA) and the Duncan test were utilized to compare normally distributed data among three or more groups. The chi-square test with Yates correction was used to compare categorical data between groups. The Pearson correlation coefficient was utilized for normally distributed data, whereas Spearman's rho was utilized for non-normally distributed data. Binary logistic regression was used to determine associations between clinical parameters and biomarkers. The logistic regression was performed as a Univariate model, and the effect of each independent variable was analyzed separately from the other variables. The logistic regression was performed using a Multiple model, where the effect of each independent variable was evaluated collectively with the other variables. The Hosmer–Lemeshow test was used to assess the reliability of the regression model. Quantitative data analysis results are shown as means ± standard deviations and medians (ranges), whereas categorical data are shown as frequencies (percentages). The significance threshold was set to *p* < 0.05.

## Results

### Demographics and clinical periodontal findings

Table 1 summarizes the study participants' demographics, including age, sex, and tooth number. There was no significant difference in sex between the periodontitis and periodontally healthy participant groups (*p* = 0.070). The periodontitis patients were significantly older than the healthy group (*p* < 0.001). The number of teeth was significantly higher in the healthy group than in the periodontitis group (*p* < 0.001). Among the periodontitis participants, 12 were diagnosed with stage III (S III) (S III GB + S III GC) periodontitis and 18 were diagnosed with stage IV (S IV) (S IV GC) periodontitis. No significant differences in sex were observed among the subgroups (healthy, S III, and S IV) (*p* = 0.120). The number of teeth was significantly higher in the healthy group than in the S IV periodontitis subgroup (*p* = 0.001).

**Table 1:** Comparison of the number of the teeth, age and gender among the healthy group and the periodontitis group, and periodontitis subgroups

	**Healthy (H) ** **(*n*=30, %50)**				**Periodontitis (S III-S IV) ** **(*n*=30, %50)**		***p***
	**Mean ± SD**	**Median (Min-Max)**			**Mean ± SD**	**Median (Min-Max)**	
No. of teeth	27.07 ± 1.23	27.5 (24 – 28)			24.93 ± 2.45	25 (20 - 28)	<0.001*
Age (years)	28.83 ± 9.95	24 (22 - 55)			40.93 ± 10.14	40.5 (19 - 65)	<0.001*
Gender							
Male	10 (33.33)				18 (60)		0.070**
Female	20 (66.67)				12 (40)		
	**Healthy (H) ** **(*n*=30, %50)**		**Stage III Periodontitis (S III) ** **(*n*=12, %20)**		**Stage IV Periodontitis (S IV) ** **(*n*=18, %30)**		***p***
	**Mean ± SD**	**Median (Min-Max)**	**Mean ± SD**	**Median (Min-Max)**	**Mean ± SD**	**Median (Min-Max)**	
No. of teeth	27.07 ± 1.23	27.5 (24- 28)^a^	25.42 ± 2.43	26.5 (22 - 28)^ab^	24.61 ± 2.48	25 (20 - 28)^b^	**0.001**†
Age (years)	28.83 ± 9.95	24 (22- 55)^b^	39.67 ± 12.25	41 (19 - 65)^a^	41.78 ± 8.74	40 (26 - 57)^a^	**<0.001**†
Gender							
Male		10 (33.3)		7 (58.3)		11 (61.1)	0.120^||^
Female		20 (66.7)		5 (41.7)		7 (38.9)	

*Mann Whitney U test, **Yates’s correction, †Kruskal Wallis H-test,^||^ Pearson chi-square test, *n* (%), a,b: Different letters represent significance. Statistical differences are marked in bold

The clinical periodontal parameters of the participants are shown in Table 2. Full-mouth PI, BOP (%), GI, PD (mm), CAL (mm), and MOB values were significantly higher in the S III, S IV, and overall periodontitis groups than in the healthy group (*p* < 0.001). CAL was significantly higher in the S IV group than in the S III group (*p* < 0.001).

**Table 2:** Comparison of clinical parameters among the healthy group and the periodontitis group, and periodontitis subgroups

	**Healthy (H) ** **(*n*=30, %50)**				**Periodontitis (S III-S IV) ** **(*n*=30, %50)**		***p***
	**Mean ± SD**	**Median (Min-Max)**			**Mean ± SD**	**Median (Min-Max)**	
Full mouth							
PI	0.22 ± 0.16	0.16 (0 – 0.6)			1.7 ± 0.62	1.57 (0.3 – 2.6)	<0.001*
BOP (%)	4.34 ± 3.14	4.16 (0 – 9.5)			54.09 ± 20.16	56.93 (17.3 - 90)	<0.001*
GI	0.25 ± 0.19	0.21 (0 – 0.8)			1.94 ± 0.51	1.84 (0.9 – 2.9)	<0.001*
PD (mm)	1.51 ± 0.16	1.54 (1.2 – 1.8)			3.3 ± 0.9	3 (1.9 – 5.7)	<0.001*
CAL(mm)	1.52 ± 0.16	1.54 (1.2 – 1.8)			4.18 ± 1.28	3.9 (2.4 – 7.7)	<0.001**
MOB	0 ± 0.01	0 (0 – 0)			0.45 ± 0.43	0.26 (0 – 1.5)	<0.001*
Sampling Site							
PI	0.09 ± 0.14	0 (0 – 0.5)			1.79 ± 0.79	1.92 (0.3 - 3)	<0.001*
BOP (%)	5.55 ± 9.11	0 (0 – 33.3)			77.22 ± 24.16	83.33 (16.7 - 100)	<0.001*
GI	0.32 ± 0.31	0.33 (0 – 1.3)			2.16 ± 0.51	2.25 (1 - 3)	<0.001*
PD (mm)	1.8 ± 0.25	1.83 (1.3 – 2.5)			5.68 ± 1.72	5.33 (2.7 – 10.3)	<0.001*
CAL(mm)	1.66 ± 0.64	1.75 (0 – 2.8)			6.53 ± 2.01	6.25 (2.8 – 11.7)	<0.001*
MOB	0 ± 0	0 (0 – 0)			0.72 ± 1.04	0.42 (0 – 5)	<0.001*
	**Healthy (H)** **(*n*=30, %50)**		**Stage III (S III)** **(*n*=12, %20)**		**Stage IV (S IV)** **(*n*=18, %30)**		***p***
	**Mean ± SD**	**Median (Min-Max)**	**Mean ± SD**	**Median (Min-Max)**	**Mean ± SD**	**Median (Min-Max)**	
Full mouth							
PI	0.22 ± 0.16	0.16 (0 – 0.6)^b^	1.52 ± 0.72	1.55 (0.3 – 2.4)^a^	1.82 ± 0.54	1.78 (0.9 – 2.6)^a^	**<0.001**†
BOP (%)	4.34 ± 3.14	4.16 (0 – 9.5)^b^	48.14 ± 16.89	50.44 (17.3 - 72)^a^	58.05 ± 21.61	60.01 (26.5 - 90)^a^	**<0.001**†
GI	0.25 ± 0.19	0.21 (0 – 0.8)^b^	1.74 ± 0.48	1.72 (0.9 – 2.7)^a^	2.07 ± 0.5	1.89 (1.1 – 2.9)^a^	**<0.001**†
PD (mm)	1.51 ± 0.16^b^	1.54 (1.2 – 1.8)	2.91 ± 0.31^a^	2.88 (2.5 – 3.4)	3.57 ± 1.07^a^	3.19 (1.9 – 5.7)	**<0.001**^||^
CAL(mm)	1.52 ± 0.16^c^	1.54 (1.2 – 1.8)	3.45 ± 0.73^b^	3.53 (2.4 – 4.6)	4.66 ± 1.36^a^	4.48 (2.9 - 7.7)	**<0.001**^||^
MOB	0 ± 0.01	0 (0 – 0)^b^	0.15 ± 0.11	0.13 (0 – 0.4)^a^	0.64 ± 0.46	0.56 (0 – 1.5)^a^	**<0.001**†
Sampling Site							
PI	0.09 ± 0.14	0 (0 – 0.5)^b^	1.58 ± 0.85	1.42 (0.3 - 3)^a^	1.94 ± 0.73	2.08 (0.7 – 2.7)^a^	**<0.001**†
BOP (%)	5.55 ± 9.11	0 (0 – 33.3)^b^	75 ± 24.09	83.33 (16.7 - 100)^a^	78.7 ± 24.79	83.33 (33.3 - 100)^a^	**<0.001**†
GI	0.32 ± 0.31	0.33 (0 – 1.3)^b^	2.15 ± 0.48	2.25 (1.3 – 2.8)^a^	2.17 ± 0.55	2.25 (1 - 3)^a^	**<0.001**†
PD (mm)	1.8 ± 0.25	1.83 (1.3 – 2.5)^b^	5.14 ± 1.03	5.17 (3 – 7.5)^a^	6.05 ± 2	5.75 (2.7 – 10.3)^a^	**<0.001**†
CAL(mm)	1.66 ± 0.64	1.75 (0 – 2.8)^b^	5.86 ± 1.17	5.58 (4.5 – 7.8)^a^	6.98 ± 2.34	6.67 (2.8 – 11.7)^a^	**<0.001**†
MOB	0 ± 0	0 (0 – 0)^b^	0.15 ± 0.23	0 (0 – 0.7)^b^	1.1 ± 1.2	0.67 (0 – 5)^a^	**<0.001**†

For binary groups, Independent Two-sample *t*-tests were used to analyze normally distributed data, whereas the Mann-Whitney U test was used for non-normally distributed data. The Kruskal-Wallis was used to compare non-normally distributed data among three or more groups. One-way analysis of variance (ANOVA) was utilized to compare normally distributed data among three or more groups.

*Abbreviations*: *PI* plaque index, *BOP* (%) bleeding on probing, *GI* gingival index, *PD* (mm) probing depth, *CAL* (mm) clinical attachment loss, *MOB* mobility

^*^Mann-Whitney U-test, ^**^Independent Samples *t*-Test, †Kruskal Wallis-H test,^||^One-way ANOVA, a-c: Different letters represent significance. Statistical differences are marked in bold

Significantly elevated PI, BOP (%), PD (mm), GI, CAL (mm), and MOB values were observed at GCF sampling sites in the periodontitis group compared with the healthy group (*p* < 0.001), similar to full mouth measurements. All periodontal parameters were significantly higher in the periodontitis groups than in the healthy group (*p* < 0.001), with the exception of MOB in the GCF sampling sites. Sampling site MOB was higher in the S IV group than in the S III and healthy groups (*p* < 0.001).

### Biochemical findings

**Fig. 1:**
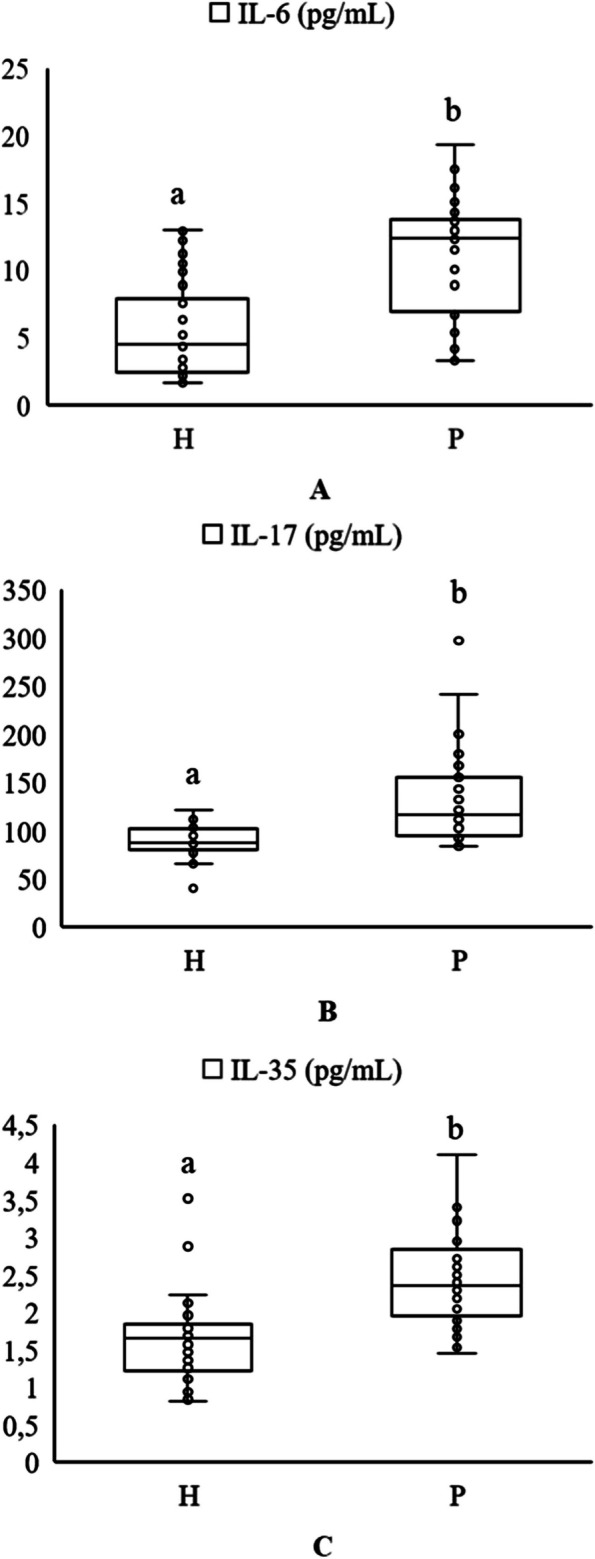
**A** Distribution of IL-6 levels in the H group and the P group. **B** Distribution of IL-17 levels in the H group and the P group. **C**. Distribution of IL-35 levels in the H group and the P group Abbreviations: H, healthy; P, periodontitis; IL, interleukin; pg, picogram; mL, milliliter. a-b: Different letters represent significance

The GCF IL-6, IL-17, and IL-35 concentrations of all groups are shown in Table 3, Fig. 1, and Additional Fig. 1. IL-6, IL-17, and IL-35 levels were significantly higher in the periodontitis group than in the healthy group (*p* < 0.001). The GCF IL-6, IL-17, and IL-35 levels were significantly higher in periodontitis subgroups than in the healthy group (*p* < 0.001). No significant differences were detected between the S III and S IV subgroups.

**Table 3:** Comparison of biochemical findings among the healthy group and the periodontitis group, and periodontitis subgroups

	**Healthy (H) ** **(*n*=30, %50)**				**Periodontitis (S III-S IV) ** **(*n*=30, %50)**		***p***
	**Mean ± SD**	**Median (Min-Max)**			**Mean ± SD**	**Median (Min-Max)**	
IL-6 (pg/mL)	5.65 ± 3.61	4.56 (1.7 - 13)			11.04 ± 4.4	12.39 (3.3 – 19.3)	<0.001^*^
IL-17 (pg/mL)	88.53 ± 17.76	87.46 (40.6 – 121.2)			130.67 ± 49.69	116.57 (83.7 - 297)	<0.001^*^
IL-35 (pg/mL)	1.63 ± 0.59	1.65 (0.8 – 3.5)			2.43 ± 0.64	2.36 (1.5 – 4.1)	<0.001^*^
	**Healthy (H)** **(*n*=30, %50)**		**Stage III (S III)** **(*n*=12, %20)**		**Stage IV (S IV)** **(*n*=18, %30)**		***p***
	**Mean ± SD**	**Median (Min-Max)**	**Mean ± SD**	**Median (Min-Max)**	**Mean ± SD**	**Median (Min-Max)**	
IL-6 (pg/mL)	5.65 ± 3.61	4.56 (1.7 – 13)^b^	10.82 ± 4.59	12.02 (4.3 – 17.6)^a^	11.18 ± 4.4	12.67 (3.3 – 19.3)^a^	<0.001**
IL-17 (pg/mL)	88.53 ± 17.76	87.46 (40.6 – 121.2)^b^	135.36 ± 60.74	116.87 (86 - 297)^a^	127.54 ± 42.42	116.57 (83.7 – 241.5)^a^	<0.001**
IL-35 (pg/mL)	1.63 ± 0.59	1.65 (0.8 – 3.5)^b^	2.17 ± 0.42	2.19 (1.5 – 2.8)^a^	2.6 ± 0.7	2.49 (1.5 – 4.1)^a^	<0.001**

*Abbreviations*: *IL* interleukin, *pg* picogram, *mL* milliliter

*Mann-Whitney U-test, **Kruskal Wallis-H test, a-b: Different letters represent significance. Statistical differences are marked in bold

### Correlations

Pearson’s/Spearman’s correlations of all data in the healthy group, periodontitis group, and S III and S IV subgroups were examined; only values with significant correlations are included in Table 4 and Additional Fig. 2.

**Table 4:** Correlations between IL-6, IL-17 and IL-35 and clinical periodontal parameters in the periodontitis group and periodontitis subgroups

**Periodontitis (S III-S IV)**		**GI**	**PD (mm)**	**SSPD (mm)**
IL-17 (pg/mL)	r	0.208*	0.395*	0.137*
	*p*	0.270*	0.031*	0.470*
IL-35 (pg/mL)	r	0.390**	0.454*	0.400*
	*p*	0.033*	0.012*	0.029*
**Stage III Periodontitis (S III)**		**SSPD (mm)**		
IL-6 (pg/mL)	r	0.634*		
	*p*	0.027*		
**Stage IV Periodontitis (S IV)**		**GI**	**PD (mm)**	**CAL (mm)**
IL-17 (pg/mL)	r	0.471*	0.629*	0.470*
	*p*	0.049*	0.005*	0.049*

Statistical differences are marked in bold

*Abbreviations*: *IL* interleukin, *GI* gingival index, *PD* (mm) probing depth, *CAL* (mm) clinical attachment loss, *SSPD* (mm) sampling site probing depth, *pg* picogram, *mL* milliliter

*Pearson correlation coefficient. **Spearman's rho correlation coefficient

IL-6 levels had a strong positive correlation with sampling site PD (r = 0.634; *p* = 0.027) in the S III subgroup.

IL-17 levels had a weak positive correlation with PD (r = 0.395; *p* = 0.031) in the periodontitis group. In the S IV subgroup, there was a strong positive correlation with PD (r = 0.629; *p* = 0.005), and moderate positive correlation with GI (r = 0.471; *p* = 0.049) and CAL (r = 0.47; *p* = 0.049).

IL-35 levels had a moderate positive correlation with PD (r = 0.454; *p* = 0.012) and sampling site PD (r = 0.4; *p* = 0.029), and a weak positive correlation with GI (r = 0.39; *p* = 0.033), in the periodontitis group.

No statistically significant correlations were observed between IL concentrations and other periodontal clinical parameters such as BOP and PI.

### Logistic regression

Associations with periodontitis were evaluated using logistic regression, and the results are presented in Table 5.

**Table 5:** Analysis of variables using logistic regression

	Groups		Univariate		Multiple	
	**Healthy (H)** (*n*=30)	**Periodontitis (S III-S IV)** (*n*=30)	OR (%95 CI)	*p*	OR (%95 CI)	*p*
Gender						
Female	10 (35.7)	18 (64.3)	Reference			
Male	20 (62.5)	12 (37.5)	3 (1.046 - 8.603)	**0.041**	24.203 (0.963 - 608.219)	0.053
Age	28.83 ± 9.95	40.93 ± 10.14	1.123 (1.054 - 1.196)	**<0,001**	1.158 (1.009 - 1.328)	**0.036**
Full mouth						
PI	0.22 ± 0.16	1.7 ± 0.62	2.725 (1.194 – 6.219)	**0.017**	---	---
Sampling site						
PI	0.09 ± 0.14	1.79 ± 0.79	4.197 (1.328 – 13.259)	**0.015**	---	---
BOP (%)	5.55 ± 9.11	77.22 ± 24.16	1.221 (1.051 – 1.418)	**0.009**	---	---
GI	0.32 ± 0.31	2.16 ± 0.51	2.147 (1.174 – 3.926)	**0.013**	---	---
Biochemistry						
IL-6 (pg/mL)	5.65 ± 3.61	11.04 ± 4.4	1.344 (1.159 – 1.56)	**<0.001**	22.327 (0.866 - 575.426)	0.061
IL-17 (pg/mL)	88.53 ± 17.76	130.67 ± 49.69	1.063 (1.025 – 1.102)	**0.001**	1.649 (1.126 - 2.415)	0.010
IL-35 (pg/mL)	16.3 ± 5.91	24.32 ± 6.35	1.261 (1.110 – 1.434)	**<0.001**	1.051 (0.978 - 1.13)	0.173

Statistical differences are marked in bold. Multiple regression model was used to adjust for the effect of age

*Abbreviations:* *IL* interleukin, *PI* plaque index, *BOP (%)* bleeding on probing, *GI* gingival index, *pg* picogram, *mL* milliliter, *n (%)*, mean± S. Deviation and median (min-max); *OR* Odds Ratio (%95 CI), *CI* Confidence Interval

According to univariate model analysis, age, male sex, full mouth PI, and sampling site PI/BOP/GI were associated with periodontitis (odds ratio [OR] = 1.123, 95% confidence interval [CI] = 1.054–1.196; OR = 3, 95% CI = 1.046–8.603; OR = 2.725, 95% CI = 1.194–6.219; OR = 4.197, 95% CI = 1.328–13.259; OR = 1.221, 95% CI = 1.051–1.418; OR = 2.147, 95% CI = 1.174–3.926, respectively) (*p* < 0.001, *p* = 0.041, *p* = 0.017, *p* = 0.015, p = 0.009, p = 0.013, respectively). Moreover, IL-6, IL-17, and IL-35 levels were associated with periodontitis (OR = 1.344, 95% CI = 1.159–1.56; OR = 1.063, 95% CI = 1.025–1.102; OR = 1.261, 95% CI = 1.110–1.434, respectively) (*p* < 0.001, *p* = 0.001, *p* < 0.001, respectively).

According to multiple model analysis, age was associated with periodontitis (OR = 1.158, 95% CI = 1.009–1.328) (*p* = 0,036). IL-35 levels were associated with periodontitis (OR = 1.649, 95% CI = 1.126–2.415) (*p* = 0.010).

## Discussion

This cross-sectional clinical study assessed the GCF concentrations of IL-6, IL-17, and IL-35 in both healthy controls and participants with severe and advanced stages (S III-S IV) of periodontitis. To our knowledge, this is the first study to report the aforementioned cytokine concentrations in GCF with regard to the periodontal disease grading system.

Interdental CAL values ≥ 5 mm at the site of greatest loss allow progression to stage S III and IV when determining the stage in the current classification system [9]. According to clinical data in the present study, CAL values significantly increased from the healthy group to the most advanced stage of the periodontitis subgroup (S IV). Although CAL values from sampling sites were significantly lower in the healthy group than in groups with periodontitis, the results were comparable for the S III and S IV subgroups. This result may be attributed to the extraction of teeth with the greatest CAL prior to measurement, as indicated by the remaining tooth number for both periodontitis subgroups. Regarding the remaining tooth number, the sole significant difference was between the healthy and S IV subgroup, consistent with the current classification (i.e., the number of teeth lost due to periodontitis is set to ≥ 5 in the S IV periodontitis subgroup when determining stage severity) [9].

According to the current classification, secondary occlusal trauma (degree of tooth mobility ≥ 2) is a local complexity feature present during progression from stage III to IV. [9] In our study, the S IV subgroup had a significantly higher sampling site MOB rate, compared with the healthy and S III groups. However, the difference between the healthy and S III groups was not statistically significant. Furthermore, the number of teeth was lower in the S IV subgroup than in the S III subgroup. Consequently, due to relatively higher tooth loss, the remaining teeth might be exposed to prominent secondary occlusal trauma, as indicated by our data.

IL-6 is a widely recognized proinflammatory cytokine that is crucial to the progression of periodontal inflammation, as well as tissue destruction [36]. Notably, the secretion of specific inflammatory mediators, (e.g., PGE2, TNF-α, IL-1β, and IL-6) is stimulated by IL-17. This stimulation ultimately activates osteoclasts, which mediate bone resorption [37–39]. In the present study, increased levels of IL-6 in both periodontitis groups suggested that these markers are directly associated with periodontal inflammation and tissue destruction; however, this significance was not maintained among periodontitis stages. Consistent with our results, several studies have revealed increased GCF levels of IL-6 in participants with periodontitis, relative to the levels in healthy controls [14, 36, 40, 41]. Except for Keleş Yücel et al., all prior research utilized the 1999 classification. Furthermore, all studies investigated the impact of this biomarker on healthy controls and participants with periodontitis, without assessing distinctions among stages.

IL-17, a proinflammatory mediator secreted by Th17 cells, is significantly increased in cases of periodontal disease. This cytokine serves a crucial function in the regulation of additional destructive cytokines [15, 16]. IL-17 also plays a significant role in bone destruction by inducing the production of RANKL [10, 42]. Previous studies showed that IL-17 secretion increases with the presence of periodontal disease [18, 43, 44]. Similarly, we found a higher GCF IL-17 level in both periodontitis subgroups. Diverse cell types are stimulated by IL-17 to secrete inflammatory biomarkers, such as IL-6, TNF-α, and IL-1 [20]. Additionally, IL-17 induces the production of IL-6 in gingival fibroblasts [45]. Based on the available data, the levels of both IL-17 and IL-6 appear to be elevated in periodontal disease, suggesting a correlation between IL-17 and IL-6 in periodontal disease. However, we did not find a positive or negative correlation between these ILs in the present study. Although there was no correlation between IL-17 and CAL, there was a positive correlation between IL-17 and PD in the periodontitis group. Because areas with increased CAL are not necessarily correlated with the deepest pockets, gingival recession could cause attachment loss in shallow pockets, where the inflammatory response is also reduced. An increase in pocket depth could be responsible for the greater GCF volume and cytokine level caused by an enhanced inflammatory reaction. Furthermore, in the S IV subgroup, the positive correlations of IL-17 with clinical values (PD, GI, and CAL) suggest that IL-17 is a pivotal cytokine involved in alveolar bone destruction in severe periodontitis cases.

In the present study, the periodontitis group exhibited considerably elevated levels of IL-35 compared with the healthy group. The levels of IL-35 did not significantly differ between the S III and S IV subgroups. The degrees of progression and distinction between the early and advanced stages of periodontitis can be determined through further studies. Although IL-35 is an anti-inflammatory agent, the elevated GCF levels in the periodontitis group could potentially be attributed to its immunosuppressive properties and serve as a protective mechanism against periodontal disease. Our results showed positive correlations of IL-35 with GI, PD, and sampling site PD in the periodontitis group, which could indicate the extent of disease progression. IL-35 is mostly synthesized by Treg cells and acts by inhibiting the inflammatory immune response [25]. Cardoso et al. assessed the frequency of Treg cells in participants with CP and healthy controls; they reported that Treg cells were significantly more numerous in the CP group [46]. The increase in IL-35 level may be related to the higher numbers of Treg cells in periodontitis. In the current literature, the correlation between IL-35 and periodontitis is not well-understood. Mitani et al. assessed IL-35 gene expression values in human gingival tissue and IL-35 concentrations in GCF [27]. Similar to our results, they observed that participants with CP had significantly higher levels of GCF IL-35 compared with healthy controls. Additionally, the gene expression levels of Epstein-Barr virus-induced gene 3 and IL-12A, which are components of IL-35, were significantly higher in the inflamed gingival tissue of participants with CP than in healthy control tissues, according to Mitani et al. [27]. Moreover, Kalburgi et al. found the highest IL-35 mRNA expression level among participants with CP, compared with the aggressive periodontitis group (stage III-IV grade C according to the 2017 classification); the level was lowest among healthy controls [47]. However, Köseoğlu et al. found higher GCF IL‑35 levels in healthy controls than in the gingivitis and CP groups [30]. The observed variations among studies indicate that IL-35, regardless of its anti-inflammatory characteristics, can serve as a protective mechanism against periodontal disease and be induced by distinct biomarker secretion mechanisms during the active and passive phases of the disease.

The present study had a few limitations. Initially, this study was designed as a cross-sectional study, with the sample size and methodology planned accordingly. The cross-sectional design prevented the evaluation of correlations and potential cause-and-effect relationships between initial and post-treatment levels of GCF ILs and periodontal health. In the future, longitudinal studies should explore comparisons across periodontitis stages and grades initially and after treatment. In our study, the control group was relatively younger compared to the periodontitis group. Initially, a univariate model was used to examine the association between the parameters. Subsequently, a multiple regression model was conducted to adjust for the effect of age on the results. It should also be taken into consideration that while designing these study groups, it was extremely difficult to find periodontally and systemically healthy participants at advanced ages, as well as to find advanced periodontitis patients at younger ages.

When constructing our study group, we selected participants with stages S III and IV periodontitis, which correspond to severe and advanced disease. Smoking, stress, hormonal changes, and diabetes disrupt the host response and influence periodontal disease pathogenesis and biomarker concentrations in oral biofluids [9]. Especially when considering grade criteria in the current classification, smoking and HbA1c level are significant modifiable risk factors in the grading system. Therefore, to make a distinct and pure comparison between ILs, systemically healthy and non-smoker controls were selected. Regarding the studied ILs, which are known indicators of the inflammatory response, we did not detect a difference between the S III and S IV periodontitis subgroups, which were stratified according to periodontitis severity. Participants in stages I and II were excluded from our study's individual cohort because they represent the initial and moderate levels in this regard.

Periodontitis is classified based on the severity and presence of periodontal breakdown and progression of disease, as well as the level of control of various risk factors, which can be supported by direct or indirect evidence [1, 9]. In the current classification, Tonetti et al. mentioned that the CAL/bone loss biomarkers-indicators section was added to the bottom of the grade table as a “gray area” that must be substantiated with specific evidence. The incorporation of particular biomarkers and their corresponding threshold values into the grade table might be substantiated by scientific evidence [9]. Our study methodology was developed with a focus on this objective. Although we included a cohort of patients with S III GB and GC, the challenge of locating systemically healthy S IV GB patients hindered our ability to construct a patient group for statistical comparison. The aforementioned challenges may be attributed to the current classification system. Creating a subgroup with slow progression characterized by a low bone loss/age ratio is challenging, even with patients aged between 18 and 65 with a CAL level of ≥ 5. We excluded patients over the age of 65 from the study, as this would disrupt the balanced match with the healthy group. Therefore, patients were selected based on these parameters to maintain the balance of the study group.

In addition to achieving the primary outcomes regarding the IL values between the healthy and periodontitis groups, our study has afforded us the opportunity to conduct an evaluation between different stages of periodontitis (Stage III and Stage IV). Overall, the study's findings suggest a significant rise in inflammatory markers such as IL-6 and IL-17 during the pathogenesis of periodontitis, indicating their strong involvement in the inflammatory process. The levels of IL-35, which serves an anti-inflammatory function, demonstrate an increase aimed at modulating inflammation within the periodontitis patients. Further investigation into these cytokines could provide valuable insights focusing on comparisons of different periodontitis stages and grades as primary aim.

## Conclusion

In conclusion, the upregulation of IL-6, IL-17, and IL-35 in individuals with S III and S IV periodontitis, along with the observed correlation between IL-17 levels and PD, GI, and CAL, suggests a potential association between severe periodontitis and these cytokines. Exploring these cytokines in more depth could yield valuable insights by focusing primarily on comparing different stages and grades of periodontitis.

### Supplementary Information

Supplementary Material 1: Additional Figure 1. A. Distribution of IL-6 levels in the H group and the periodontitis subgroups. B. Distribution of IL-17 levels in the H group and the periodontitis subgroups. C. Distribution of IL-35 levels in the H group and the periodontitis subgroups.

Supplementary Material 2: Additional Figure 2. A. The correlations between clinical periodontal measurements and the biochemical findings in the periodontitis group. B. The correlations between clinical periodontal measurements and the biochemical findings in the S III group. C. The correlations between clinical periodontal measurements and the biochemical findings in the S IV group.

## Data Availability

The data that support the findings of this study are available from the corresponding author upon reasonable request.

## Abbreviations

IL: Interleukin
OR: Odds ratio
CI: Confidence interval
Th17: T helper 17
GI: Gingival index
PI: Plaque index
PD: Pocket depth
CAL: Clinical attachment loss
BOP: Bleeding on probing
MOB: Mobility
GCF: Gingival crevicular fluid
TNF: Tumor necrosis factor
CP: Chronic periodontitis
RANKL: Receptor activator of nuclear factor-κ B ligand
IU: Istanbul University
FD: Faculty of Dentistry
ELISA: Enzyme-linked immunosorbent assays
PBS: Phosphate-buffered saline
S III: Stage III
S IV: Stage IV
