# Supplementary material for: Interleukin-6, -17, and -35 levels in association with clinical status in stage III and stage IV periodontitis: a cross-sectional study
Source: BMC Oral Health. 2024 Aug 30;24:1015. doi: 10.1186/s12903-024-04751-3 (PMC11363592; doi:10.1186/s12903-024-04751-3)
Supplement: Supplementary file 1 — Supplementary Material 1: Additional Figure 1. A. Distribution of IL-6 levels in the H group and the periodontitis subgroups. B. Distribution of IL-17 levels in the H group and the periodontitis subgroups. C. Distribution of IL-35 levels in the H group and the periodontitis subgroups. [file 12903_2024_4751_MOESM1_ESM.docx]

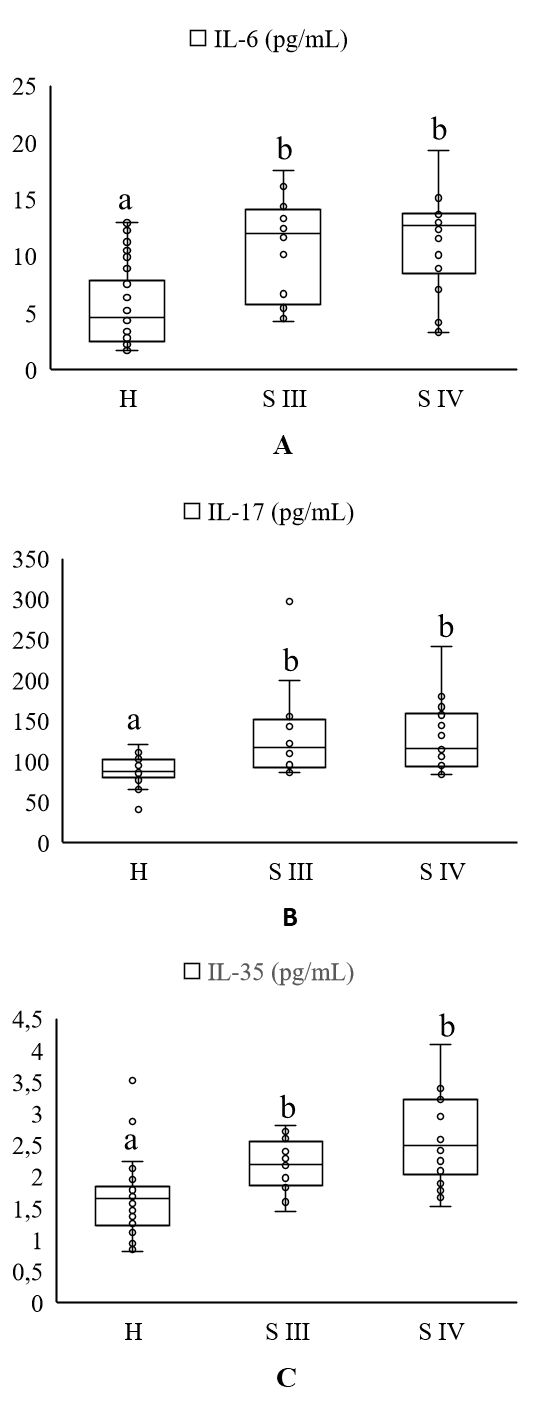


Additional Figure 1. **A**. Distribution of IL-6 levels in the H group and the periodontitis subgroups. **B**. Distribution of IL-17 levels in the H group and the periodontitis subgroups. **C**. Distribution of IL-35 levels in the H group and the periodontitis subgroups.

Abbreviations: H, healthy; S III, Stage III periodontitis; S IV, Stage IV periodontitis; IL, interleukin; pg, picogram; mL, milliliter.

a-b: Different letters represent significance.
