# Supplementary material for: Interleukin-6, -17, and -35 levels in association with clinical status in stage III and stage IV periodontitis: a cross-sectional study
Source: BMC Oral Health. 2024 Aug 30;24:1015. doi: 10.1186/s12903-024-04751-3 (PMC11363592; doi:10.1186/s12903-024-04751-3)
Supplement: Supplementary file 2 — Supplementary Material 2: Additional Figure 2. A. The correlations between clinical periodontal measurements and the biochemical findings in the periodontitis group. B. The correlations between clinical periodontal measurements and the biochemical findings in the S III group. C. The correlations between clinical periodontal measurements and the biochemical findings in the S IV group. [file 12903_2024_4751_MOESM2_ESM.docx]

Supplementary Figure 2. **A**. The correlations between clinical periodontal measurements and the biochemical findings in the periodontitis group. **B**. The correlations between clinical periodontal measurements and the biochemical findings in the S III group. **C**. The correlations between clinical periodontal measurements and the biochemical findings in the S IV group.

Abbreviations: IL, interleukin; PI, plaque index; BOP (%): bleeding on probing; GI, gingival index; PD (mm), probing depth; CAL (mm), clinical attachment loss; MOB; mobility; SSPI, sampling site plaque index; SSBOP (%): sampling site bleeding on probing; SSGI, sampling site gingival index; SSPD (mm), sampling site probing depth; SSCAL (mm), sampling site clinical attachment loss; SSMOB; sampling site mobility; pg, picogram; mL, milliliter.

Green colour represents positive correlation. Red colour represents negative correlation.
